# L'esperienza di uno specializzando

**DOI:** 10.1186/1824-7288-41-S2-A58

**Published:** 2015-09-30

**Authors:** Andrea Pietravalle

**Affiliations:** 1 Residency School in Pediatrics of the University of Rome “La Sapienza”, Rome, Italy

## 

Located in the central plateau area of Tanzania in East Africa, St Gaspar's Hospital was founded in 1968 by the Society of the Precious Blood Catholic Missionaries.

Elevated to referral hospital at a regional level, with an approved capacity of 320 beds, it provides residential consultancy services in general medicine, surgery, obstetrics, gynecology and pediatrics for more than 100,000 people.

In 2013, a cooperation agreement between the “Bambino Gesù” Children's Hospital (OPBG) and St Gaspar's has been established for the creation of a “Clinical and Surgical Pediatric Centre”. That led to the complete renovation of the Pediatric Ward, the construction of a new operating theatre and the installation of a telemedicine system for remote medical diagnosis and consultation.

In this setting, a periodic turnover of clinical and surgical missions is guaranteed in order to form the local healthcare providers and improve the quality of assistance.

From 2014, the Residency School in Pediatrics of the University of Rome “La Sapienza”, in agreement with OPBG, allows its residents to spend a 3-month period of work at the St Gaspar's Hospital's Pediatric Ward.

Under the supervision of a local tutor, residents are involved in clinical activities including: morning work round with 90 to 150 patients visited per day; evaluation and management of new admissions for malaria, typhoid fever, meningitis, gastroenteritis, severe malnutrition and local herbs intoxication; weekly mobile clinic service, for antenatal visits, vaccinations and health education programs in rural areas around the hospital. Residents are also engaged in research activities and training courses for local health workers.

One year has passed from when I decided to join the OPBG project but memories and feelings of those days are still vivid in my mind. From the sorrow for the premature deaths that you know easily preventable in developed countries to the joy for the smile of the baby you manage to treat and send back home. In the end, what you receive from this kind of experience is so much more than what you give and that is why I am certain to say, “I want to do it again”.

